# The future of critical care: renal support in 2027

**DOI:** 10.1186/s13054-017-1665-6

**Published:** 2017-04-11

**Authors:** William R. Clark, Mauro Neri, Francesco Garzotto, Zaccaria Ricci, Stuart L. Goldstein, Xiaoqiang Ding, Jiarui Xu, Claudio Ronco

**Affiliations:** 1 School of Chemical Engineering, Purdue University, 480 Stadium Mall Drive; FRNY 1051, West Lafayette, IN 47907 USA; 2 International Renal Research Institute of Vicenza (IRRIV), San Bortolo Hospital, Vicenza, Italy; 3 Department of Cardiology and Cardiac Surgery, Pediatric Cardiac Intensive Care Unit, Bambino Gesù Children’s Hospital, IRCCS, Rome, Italy; 4 Center for Acute Care Nephrology, Cincinnati Children’s Hospital Medical Center, Cincinnati, OH USA; 5 Department of Nephrology, Zhongshan Hospital, Fudan University, Shanghai, China; 6 Shanghai Institute of Kidney Disease and Dialysis, Shanghai Quality Control Center for Dialysis, Shanghai, China; 7 Department of Nephrology, San Bortolo Hospital, Vicenza, Italy

## Abstract

Since its inception four decades ago, both the clinical and technologic aspects of continuous renal replacement therapy (CRRT) have evolved substantially. Devices now specifically designed for critically ill patients with acute kidney injury are widely available and the clinical challenges associated with treating this complex patient population continue to be addressed. However, several important questions remain unanswered, leaving doubts in the minds of many clinicians about therapy prescription/delivery and patient management. Specifically, questions surrounding therapy dosing, timing of initiation and termination, fluid management, anticoagulation, drug dosing, and data analytics may lead to inconsistent delivery of CRRT and even reluctance to prescribe it. In this review, we discuss current limitations of CRRT and potential solutions over the next decade from both a patient management and a technology perspective. We also address the issue of sustainability for CRRT and related therapies beyond 2027 and raise several points for consideration.

## Background

Continuous renal replacement therapy (CRRT) was developed originally as an alternative for hemodynamically unstable acute renal failure (ARF) patients who could not tolerate conventional hemodialysis [1, 2]. The early application of CRRT largely involved technology adapted from the maintenance dialysis setting and almost exclusively occurred as a salvage therapy, typically in hypercatabolic patients with severe, diuretic-resistant fluid overload. As CRRT technology evolved over the past four decades to produce devices specifically designed for the critically ill population, utilization of the therapy as a first-line treatment for acute kidney injury (AKI) and the patient populations treated have expanded substantially [3]. Moreover, despite a lack of data definitively showing outcome benefits for CRRT, consensus statements now suggest its use, rather than conventional hemodialysis, for hemodynamically unstable AKI patients [4].

While CRRT is now a mainstay therapy in the vast majority of large ICUs around the world, this modality can be challenging to implement at some institutions and significant opportunity for improvement exists [5]. Clinicians’ uncertainty about numerous aspects of CRRT, including therapy dosing, timing of initiation and termination, fluid management, anticoagulation, drug dosing, and data analytics, may lead to inconsistent delivery of the therapy and even reluctance to prescribe it. In this review, we perform a critical assessment of CRRT, discussing current limitations and potential solutions over the next decade from both a patient management and a technology perspective. In addition, we address the issue of sustainability for CRRT and related therapies beyond 2027 and raise several points for consideration.

## Renal support in 2027: addressing CRRT’s current limitations

### Adoption of precision CRRT

In the most recent Acute Dialysis Quality Initiative (ADQI) consensus conference, the participants rightly identified the need for CRRT patient management to align with the current focus on personalized medicine. In this regard, the ADQI participants proposed the term “precision CRRT” in calling for technology to be applied on an individualized basis [6] rather than a “one size fits all” approach that is all too common in current practice. An important component of this individualized approach has been termed “dynamic CRRT”, in which the treatment is adapted to the constantly changing clinical status of the critically ill AKI patient [7]. In addition to ongoing clinical assessment of the patient, important technical components of dynamic CRRT include solute clearance, delivered/prescribed dose, effective treatment time, solute control indicators, circuit/filter pressure trends, fluid and hemodynamic management, and anticoagulation. Also assuming important roles in the implementation of precision CRRT are quality metrics [8], biofeedback [9], and data analytics [10], all of which are discussed further in the following.

### Dosing of CRRT

Based on the landmark study performed by Ronco et al. [11] and several other prospective trials [12–16], the use of effluent-based dosing to guide CRRT prescription and delivery is established firmly in clinical practice. Nevertheless, the effluent dose (expressed as ml/kg/hr) does not provide an accurate estimation of actual solute clearance and considerable confusion exists among clinicians, especially those familiar with urea-based dose measurements in the maintenance dialysis setting. Indeed, substantial differences between the effluent dose and actual solute clearance may exist under many CRRT operating conditions [17]. Therefore, we recently reappraised dose prescription and delivery for CRRT [18], and proposed an adaptation of a chronic dialysis parameter (standard Kt/V) [19] as a benchmark to supplement effluent-based dosing. Our proposal allows for the target standard Kt/V to vary on a patient-to-patient basis according to clinical circumstances and can be modified in an individual patient, depending on the clinical course (e.g., a hypercatabolic, septic patient in need of higher dose to control azotemia). These dosage adjustments are entirely consistent with the concept of dynamic CRRT.

The clinical relevance of urea as a toxin per se is very much an open question, especially in light of large prospective studies performed in patients with end-stage renal disease (ESRD), and many experts give credence to the potential importance of other uremic toxin classes [20, 21]. However, identification of a larger molecular weight toxin which is easily measured in clinical practice and has well understood kinetic properties for different renal replacement therapies has been elusive [22]. Because the current reality is that urea is the only surrogate molecule whose kinetics during renal replacement therapy are well understood, we believe our proposal for application of standard Kt/V to CRRT is rational.

For the future, we believe the incorporation of effluent urea nitrogen measurements, first through clinical protocols [23] and then CRRT machines equipped with online sensors [24], will occur in clinical practice. In addition, machines will provide clinicians with automated alerts when therapy trends suggest filter clotting, based on changes in effluent measurements or circuit pressures. Moreover, we predict that additional molecules, having specific relevance for AKI pathophysiology [25, 26], will be validated by 2027 as CRRT dose surrogates for patients with AKI and other disorders.

### Timing of CRRT initiation

Recent data have cast doubt on the use of conventional ESRD-based criteria for RRT initiation in AKI patients [27]. Nevertheless, decisions about initiation of RRT for AKI continue to be difficult due to the lack of a clinically relevant parameter that has been validated in prospective trials. Moreover, recent prospective trials employing different initiation criteria have provided conflicting results [28, 29]. In a recently completed pilot trial from Canada, Wald et al. [30] demonstrated the validity of changes in urine output, whole-blood NGAL concentration, and serum creatinine as initiation criteria—a full-scale RCT is now being performed.

We believe the concept of demand/capacity imbalance, proposed recently by Mehta and colleagues [31, 32], will be validated in clinical trials as a useful parameter guiding decisions about CRRT initiation and incorporated into clinical practice by 2027. The components of renal demand include AKI disease burden, solute load, and fluid load. A significant imbalance between this demand and diminished renal function in AKI patients should prompt serious consideration of RRT initiation.

The concept of demand/capacity balance will also be useful to guide decisions about renal recovery and RRT cessation. The ADQI group has recommended explicitly that RRT should be discontinued if kidney function has recovered sufficiently to reduce the demand–capacity imbalance (current and expected) to acceptable levels [32]. We believe the preliminary work defining the important considerations with respect to renal recovery [33, 34] will be refined over the next decade, allowing clinicians to make more informed decisions about RRT termination.

While awaiting the results from clinical trials, we also believe further progress will be made by 2027 in the clinical application of biomarkers [35, 36], not only for the initial diagnosis of AKI but also for decisions about CRRT initiation and termination. In addition, progress will be made in validating real-time GFR measurements for both of these applications [37]. We predict that these technologies will be used routinely in conjunction with established clinical criteria, especially the extent of fluid overload (see later), to guide CRRT initiation. Likewise, these technologies will be useful in decisions for CRRT discontinuation or transition to another modality.

### Management of fluid overload

Severe fluid overload continues to be a common trigger for CRRT initiation, especially in septic shock patients who have received aggressive volume resuscitation in the face of worsening renal function [38]. A quantitative parameter of fluid accumulation, percent fluid overload (%FO), has been employed in many recent clinical trials [39, 40] and positive values have been associated with increased mortality, especially when >10% at RRT initiation. In addition to septic AKI patients, postsurgical patients are also at high risk of developing severe fluid overload once AKI develops. Xu et al. [41] found that a cumulative %FO ≥ 7.2% had a significant impact on 90-day outcome in critically ill AKI patients after cardiac surgery.

Even while approaches designed to assess fluid overload and quantify fluid responsiveness are refined [42], we predict that the occurrence of fluid overload as the primary trigger for CRRT initiation will increase over the next decade. The basis for this belief relates to the demographics of severe AKI, for which the primary cause will increasingly be severe sepsis and septic shock during this time. Thus, in conjunction with %FO (or similar measurement) and other clinical parameters, technologies providing real-time fluid assessment capabilities, including bioimpedance and ultrasound, will be used routinely by 2027 [43].

### Antibiotic dosing during CRRT

The increased prevalence of sepsis-associated severe AKI over the next decade will result in the need for antibiotic therapy in an increasingly greater percentage of CRRT patients [44]. The lack of reliable clinical data to guide antibiotic use and the associated risk of under-dosing during CRRT have been identified as major problems—dosing continues to be done largely on an empiric basis [45] (Table 1). Over the next decade, multiple clinical trials evaluating the antibiotics most commonly prescribed to CRRT patients will be performed. The typical range of CRRT flow parameters will be evaluated in these trials, along with commonly used filters, so that the contributions of diffusive, convective, and adsorptive clearance can be ascertained. These trials will provide relatively precise dosing recommendations for a series of widely used antibiotics, leading to the routine incorporation of this information into the CRRT prescription by clinicians.

**Table 1:** Proposed elements for CRRT pharmacokinetic assessment

• Estimation of pharmacokinetic parameters, including variability	
• Comparison of pharmacokinetic parameters with those of typical patients with normal kidney function (literature or sponsor data) or the appropriate reference population	
• Quantification of the impact of changes in the prescribed Q_e_ on the pharmacokinetic parameters of interest, and interpolation for flow rates not evaluated in this study	
• Assessment of whether dosage adjustment is warranted in CRRT recipients	
• If dosage adjustment is warranted, derivation of specific dosing recommendations for the studied conditions	

Reprinted with permission from [45]

*CRRT* continuous renal replacement therapy

Q_e_ - effluent rate (ml/hr)

### Anticoagulation

A series of recent prospective trials have demonstrated that regional citrate anticoagulation (RCA) significantly reduces the risk of hemorrhage for patients treated with CRRT (in comparison with heparin) [46]. The majority of these recent studies have involved physiologic (as opposed to hypertonic) citrate solutions, allowing them to serve as both anticoagulant and replacement solutions. Moreover, some of these studies involved machines capable of semi-automated RCA delivery in which citrate infusion rates are modulated by device software, at least to some extent [47]. We predict that CRRT machines will provide more fully automated RCA by 2027, as has been proposed for other acute RRT modalities [48]. Finally, we foresee that the use of heparin as an anticoagulant for CRRT will be markedly reduced by 2027, due to its hemorrhagic risks in the CRRT population.

In parallel with advances in RCA, we believe manufacturers will continue in the pursuit of developing antithrombogenic membranes that either minimize or obviate the requirement for anticoagulation during CRRT. Surface-modified versions of the AN69 membrane [49] have been developed but clinical data demonstrating acceptable circuit lives during CRRT performed with no anticoagulation are currently lacking.

Finally, another issue related to thrombogenicity during CRRT is from the perspective of catheter use. Catheter thrombosis is a very common treatment complication, resulting in decreased therapy delivery and contributing to significant morbidity and cost. Recent data suggest that use of a surface-modified catheter (in comparison with a standard unmodified polyurethane catheter) results in a longer catheter life and less dysfunction (as measured by blood flow rate) [50]. We believe further progress in the development of surface-modified catheters will occur over the next decade, leading to less catheter-related dysfunction and higher blood flow capabilities.

### Quality metrics

One of the current factors potentially limiting outcome improvements and further dissemination of the therapy is the lack of standardization for CRRT. A specific limitation that contributes to this lack of standardization is an insufficient evidence base—the current confusion regarding the timing of CRRT initiation is a good example. As such, we predict that both randomized and pragmatic clinical trials performed over the next decade will address such critical issues and improve therapy standardization.

Another major factor limiting therapy standardization is the lack of consensus CRRT quality metrics. Rewa et al. [8] are currently evaluating which aspects of CRRT prescription and delivery should be targets for quality metric development, namely dose (including treatment downtime), anticoagulation, vascular access, and circuit-related issues (Table 2). As is the case in maintenance dialysis, we predict that a number of these quality metrics will be established by consensus initiatives and be part of routine clinical practice in 2027. Another recent development which will support therapy standardization is the use of simulation-based CRRT training, which has demonstrated tangible improvements in the delivery of CRRT [51].

**Table 2:** Proposed quality metrics for CRRT

Theme	Measures
Dose prescription	High vs low dose
Dose delivery	Percentage of prescribed dose delivered
Anticoagulation selection	Heparin vs citrate vs none
Anticoagulation monitoring	PTT monitoring, citrate monitoring
Anticoagulation complications	Bleeding, hypocalcemia, incidence of HIT
Treatment interruption	Number of interruptions and duration of interruptions; time to establish new circuit
Catheter-related issues	Infections, bleeding, obstruction/thrombosis
Circuit-related issues	Hiter clotting, pressure alarming

Reprinted with permission from [8]

*CRRT* continuous renal replacement therapy, *PTT* partial thromboplastin time, *HIT* heparin-induced thrombocytopenia

### CRRT data analytics and biofeedback

**Fig. 1:**
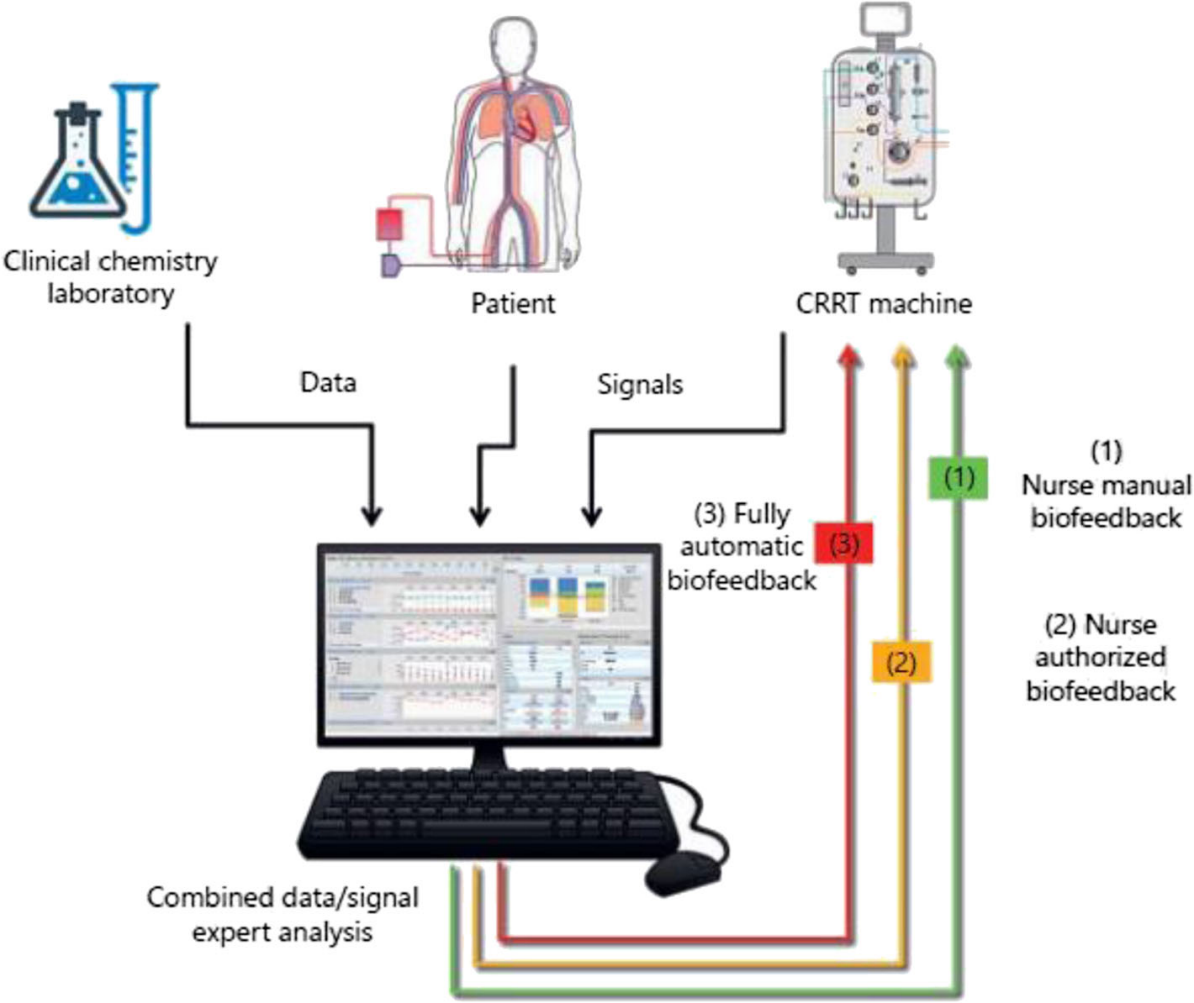
Various approaches for biofeedback in CRRT. Reprinted with permission from [52]. *CRRT* continuous renal replacement therapy

The technical limitations of current CRRT machines make efficient management of patient and treatment data difficult in some respects [10]. As opposed to the automated, real-time data capture that characterizes many interventions in the ICU, CRRT machine data generally are collected and analyzed manually at present. This is a laborious, time-consuming process that frequently delays necessary treatment intervention and is a barrier to providing dynamic CRRT. A desired technical aspect of dynamic CRRT is the availability of real-time CRRT machine data as part of a biofeedback system. While any prescription changes needed to close a biofeedback loop have to be made manually by the clinical team at present, we predict that such changes will be made automatically by the CRRT machine in 2027 [52] (Fig. 1). This will be accomplished by the incorporation of online tools for continuous, real-time measurement of dose delivery and fluid overload. Moreover, in addition to treatment data from the CRRT machine, patient-level data from the electronic medical record (EMR) will play a critical role in these biofeedback loops [53].

A dynamic CRRT program also implies the ability to use information technology beyond the real-time phase for longer-term purposes. At present, CRRT machine data are not routinely stored in an accessible warehouse, rendering impossible the systematic generation of reports for review by the clinical team. We predict that by 2027 clinicians will routinely be able to assess historical trends on a facility-level basis, especially those related to the basic quality metrics mentioned earlier, or to use these data for quality assurance purposes. Moreover, these data will facilitate design and implementation of pragmatic trials, including registries. Again, patient-level data from the EMR will supplement technical data.

### Extracorporeal multiorgan support

**Fig. 2:**
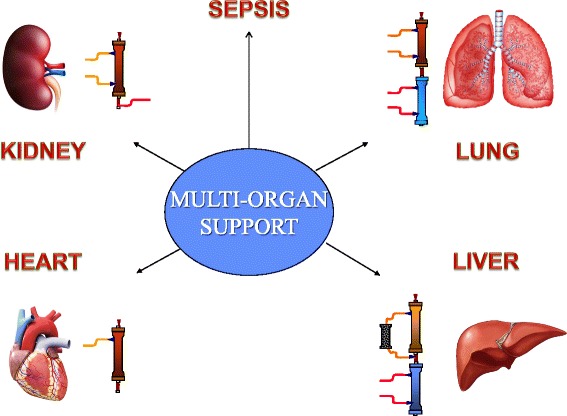
Components of extracorporeal multiorgan support

While several extracorporeal approaches have been used as adjunctive therapies for organ failures beyond AKI [49, 54–60], additional clinical outcome data are clearly needed. We believe prospective trials performed over the next decade will demonstrate outcome benefits for such approaches as adjunctive therapies (Fig. 2). We feel that extracorporeal systems designed to eliminate CO_2_ (as an adjunct to low tidal volume ventilation) [54] and modulate inflammatory mediators (as an adjunct to sepsis therapy) have the greatest likelihood of showing these benefits. With regard to sepsis adjunctive therapies, we predict that mediator modulation will be achieved through both filter-based [57, 58] and hemoperfusion [59, 60] techniques.

### Miniaturization of technology

As is the case with data analytics and information management, CRRT is lagging behind many other therapies with respect to technology “down-sizing”. The incorporation of microfluidics, micromechanics, and nanotechnology is driven by the desire not only to decrease the physical footprint of medical technologies (thus enhancing portability) but also to extend their applicability to greater numbers and subsets of patients [61]. We predict that the enhanced portability of future devices will allow for a set of similar devices to be used over the entire RRT spectrum (ICU, ward, and even home) in a given patient. In turn, this will allow for more seamless transitions in care, leading to increased simplicity and possibly lower costs.

One clear example of this trend within the renal replacement field is the development of wearable dialysis and ultrafiltration devices, on which several investigative groups have made significant progress during the past decade [62, 63]. While the initial application of these devices has largely been focused on ESRD patients, they may yet prove useful in the management of fluid overload, especially in the setting of heart failure. Another potential application in the future is their use for severe AKI survivors who need supplementation of kidney function in the recovery phase.

The recent development of a CRRT device specifically designed to treat pediatric AKI patients is a more immediate-term example of technology miniaturization. While treatment of pediatric AKI with CRRT has grown substantially over the past decade [64], pediatricians have been forced to use equipment designed primarily for adult patients. The design features of traditional CRRT equipment, especially with regard to fluid accuracy of the machine and extracorporeal blood volume of the circuit, have rendered pediatric treatments problematic—this is especially true for neonatal patients, who typically weigh <3 kg. Based on the recent success reported by Ronco et al. in the management of neonatal AKI with the CARPEDIEM device [65], we predict further growth in the utilization of CRRT for pediatric AKI will occur over the next decade. Furthermore, we believe the technology advances that made the CARPEDIEM device possible will accelerate the application of miniaturization principles to other aspects of both pediatric and adult CRRT.

### Nondialytic management of AKI

The need for efficient processing of data in the management of critically ill AKI patients has been highlighted during a recent ADQI conference focused on the implications of “big data” for this population [66]. While the ability to utilize data efficiently is a challenge during CRRT, limitations also exist upstream with regard to AKI diagnosis. Moreover, recent data also demonstrate that postdischarge management of patients who have had an AKI episode is fragmented and unpredictable [67]. While early studies evaluating “sniffers” and alerts designed to facilitate the diagnosis of AKI have provided conflicting results [68], we predict that their utility will be demonstrated conclusively in prospective trials and they will become part of standard clinical practice by 2027. Likewise, over the next decade, web-based algorithms will be developed to triage post-AKI follow-up to nephrologists or other medical specialties according to AKI severity, extent of CKD, and comorbidities. These algorithms will be used routinely in clinical practice to minimize the risk of progression to ESRD.

### Health economics

The cost of medical care for both chronic diseases and acute conditions is increasingly being scrutinized by government agencies and payers, in both the developed and the developing world. Because the societal costs of treating relatively small numbers of patients with ESRD are disproportionately high, health economic analyses and cost-effectiveness studies are now performed frequently upon the introduction of new chronic dialysis therapies. While visibility for the costs of treating AKI seems to be low and few health economic assessments have been performed, it is a costly disorder [69, 70]. Recent health economic assessments have demonstrated that simplistic comparisons of only product costs for different AKI renal replacement modalities are not informative, because they capture only a very short-term time window. Valid comparisons instead have to incorporate the costs of long-term outcomes potentially related to modality choice, including ESRD [71]. We predict that more rigorous health economic analyses will be performed in the AKI setting over the next decade and robust assessments will be required on a routine basis in 2027 for new technologies [72].

Another important consideration is the general affordability of acute RRT in the future. In the developed world, acute RRT costs typically are not an important factor due to widespread health insurance coverage and relatively generous reimbursement policies. On the other hand, hospital reimbursement and patient self-payment policies vary considerably across the developing world [73], resulting in the need for some patients and their families to make very difficult decisions about potentially life-saving medical technology. We believe an increased demand for CRRT in the developing world will be satisfied by a combination of expanded insurance coverage and lower overall cost of CRRT delivery in the future.

## Renal support in 2027: sustainability of dialysis techniques

A final consideration over the next decade is the need to begin developing acute dialysis techniques designed to provide sustainability beyond 2027. One of the major environmental footprints associated with most extracorporeal dialysis modalities is the generation of large masses of plastic disposables, including filters and tubing sets. The development of miniaturized technologies, including wearable devices, would be an important advance in addressing this problem. Another important dialysis-related environmental footprint is the large volume of fluid generated as waste [74]. Both sorbent-based techniques [61, 62, 75] and membrane-based reclamation of spent fluid [76] are potential approaches for reducing the volume of effluent generated during CRRT and other dialysis modalities. We predict that progress in addressing long-term sustainability for acute renal support modalities will begin to be made over the next decade.

## Conclusion

As the utilization of CRRT and ancillary therapies in the management of critically ill patients increases, their limitations have become more evident to many clinicians. These limitations apply both to the manner in which patients are clinically managed and how the technology is used. We have critically assessed these problems and rendered predictions about the manner in which they will be solved over the next decade. While we have tried to prognosticate on several topics, we have not attempted to address certain contentious issues that we believe will continue to be debated even in 2027. For example, we believe questions about the optimal CRRT mode [77] and nutritional regimen [78] will not be resolved completely. We have also attempted to raise awareness about the issue of sustainability for CRRT beyond 2027 and have raised several points for consideration. While a number of problems currently exists, we believe the future of extracorporeal therapy for critically ill patients is very bright and its use in this patient population will continue to grow.

## Abbreviations

%FO: Percent fluid overload
ADQI: Acute Dialysis Quality Initiative
AKI: Acute kidney injury
CKD: Chronic kidney disease
CO_2_: Carbon dioxide
CRRT: Continuous renal replacement therapy
EMR: Electronic medical record
ESRD: End-stage renal disease
GFR: Glomerular filtration rate
ICU: Intensive care unit
RCA: Regional citrate anticoagulation
RCT: Randomized controlled trial
RRT: Renal replacement therapy
